# Opportunities and short-comings of the axolotl salamander heart as a model system of human single ventricle and excessive trabeculation

**DOI:** 10.1038/s41598-022-24442-9

**Published:** 2022-11-28

**Authors:** Sophie Meyer, Henrik Lauridsen, Kathrine Pedersen, Sofie Amalie Andersson, Pim van Ooij, Tineke Willems, Rolf M. F. Berger, Tjark Ebels, Bjarke Jensen

**Affiliations:** 1 Center for Congenital Heart Diseases, Department of Cardiothoracic Surgery, University Medical Center Groningen, Groningen, The Netherlands; 2 Department of Clinical Medicine, Aarhus University, Aarhus N, Denmark; 3 Department of Radiology and Nuclear Medicine, Amsterdam University Medical Centers, location AMC, Amsterdam, The Netherlands; 4 Department of Radiology, University Medical Center Groningen, Groningen, The Netherlands; 5 Center for Congenital Heart Diseases, Department of Pediatric Cardiology, University Medical Center Groningen, Groningen, The Netherlands; 6 Department of Medical Biology, Amsterdam Cardiovascular Sciences, Amsterdam University Medical Centres, Meibergdreef 15, 1105AZ Amsterdam, The Netherlands

**Keywords:** Cardiovascular biology, Physiology, Cardiology, Animal physiology

## Abstract

Few experimental model systems are available for the rare congenital heart diseases of double inlet left ventricle (DILV), a subgroup of univentricular hearts, and excessive trabeculation (ET), or noncompaction. Here, we explore the heart of the axolotl salamander (*Ambystoma mexicanum*, Shaw 1789) as model system of these diseases. Using micro-echocardiography, we assessed the form and function of the heart of the axolotl, an amphibian, and compared this to human DILV (n = 3). The main finding was that both in the axolotl and DILV, blood flows of disparate oxygen saturation can stay separated in a single ventricle. In the axolotl there is a solitary ventricular inlet and outlet, whereas in DILV there are two separate inlets and outlets. Axolotls had a lower resting heart rate compared to DILV (22 vs. 72 beats per minute), lower ejection fraction (47 vs. 58%), and their oxygen consumption at rest was higher than peak oxygen consumption in DILV (30 vs. 17 ml min^−1^ kg^−1^). Concerning the ventricular myocardial organization, histology showed trabeculations in ET (n = 5) are much closer to the normal human setting than to the axolotl setting. We conclude that the axolotl heart resembles some aspects of DILV and ET albeit substantial species differences exist.

## Introduction

Human functionally univentricular hearts are characterized by only one fully developed ventricle^1^. The other ventricle is hypoplastic or, rarely, is completely absent^2^. Most of the systemic venous return as well as pulmonary venous return pass through the same ventricle without anatomical separation^3^. There is no cure and only palliation is possible. Most patients are currently palliated with a sequence of operations during their neonatal and childhood phase culminating in the Fontan circulation. This entails the creation of a direct connection between the systemic venous return and the pulmonary arteries, bypassing the heart^4^. Fontan patients suffer from the long-term consequences of this un-physiological circulation, which severely limits their quality of life and often leads to an untimely death^5, 6^. Some patients with functionally univentricular hearts live without the standard Fontan palliation^7–9^. Long-term survival is probably related to favorable intraventricular streaming of blood flows with disparate oxygen saturation and a pulmonary artery stenosis^10^.

Functionally univentricular hearts resemble the setting in the amphibians, where there is a single ventricle only^11–13^. The axolotl salamander (*Ambystoma mexicanum* Shaw 1789) represents the oldest lab animal in a self-sustained population and is a widely used model in the fields of evolutionary developmental biology and regenerative biology^14^. The axolotl ventricular cavity is not only univentricular, but contains a very high number of trabeculations just as in other cold-blooded vertebrates^15^. In human, an excessively trabeculated ventricular wall allows for the diagnosis of ventricular noncompaction cardiomyopathy and it can afflict either or both ventricles^16^. Noncompaction is the most commonly used term for this affliction, but it presumes an etiology that is disputed whereas ‘excessive trabeculation’ (ET), which we will use henceforth, describes the setting irrespective of the underlying etiology^17^. So-called true cases are characterized by poor pump function^18^.

Functionally univentricular heart and ET are rare congenital heart diseases for which almost no valid experimental model systems exist. For the first time, the axolotl will serve as a model to deepen our understanding of the human univentricular heart and its functional anatomy and hemodynamics as well as deepen our understanding of ET. The aim of this study is to characterize heart function and structure of the axolotl hearts in comparison to human cases of DILV and ET.

## Material and methods

### Axolotl

#### Animals

In total, fifteen axolotls, body mass (BM) = 39.2 ± 25.2 g, total length (TL) = 17.2 ± 4.5 cm, of different color variants (brown/green wild type, leucistic, albino, red) and sex were used in the experiments (Table 1). Animals were housed individually in chloride and chloramines free tap water in 10 l or 20 l plastic containers with a weekly water change on a 12 h/12 h light/dark cycle and fed every other day with axolotl pellets. Housing was in accordance with Danish law and experiments were conducted under The Danish Animal Experiments Inspectorate protocol #2015-15-0201-00615. All experiments were performed in accordance with relevant named guidelines and regulations. The authors complied with the ARRIVE guidelines. In addition, for the comparison of ventricular wall anatomy we re-examined histological sections of *Xenopus* hearts that have previously been published^19^.

**Table 1:** Characteristics and measured physiological parameters of the used axolotls.

Axolotl	Group A: Heart modeling/echo injections	Group B: Heart function	Group C: Evans blue injection	Group D: Oxygen consumption
Number of animals in group	3	4	2	6
Body mass (g)	17.2 ± 6.3	35.1 ± 4.0	6.6 ± 0.28	63.7 ± 17.3
Total length (cm)	13.0 ± 1.6	18.4 ± 0.3	9.8 ± 0.4	21.0 ± 2.3
Heart rate (beats/min)	27.3 ± 6.4 (propofol)	43.3 ± 0.5 (benzocaine)	47.5 ± 6.4 (propofol)	21.7 ± 4.1 (resting, unanesthetized)
Ventricle volume (end diastole) (µl)		130.5 ± 66.3		
Ventricle volume (end systole) (µl)		89.3 ± 57.5		
Stroke volume (µl)	33.1 ± 13.1	41.3 ± 8.5		
Mass specific cardiac output (ml min^−1^ kg^−1^)	52.3 ± 13.7	51.6 ± 14.3		
Ventricle surface area (end diastole) (mm^2^)		193.0 ± 64.4		
Ventricle surface area (end systole) (mm^2^)		154.8 ± 68.8		
Ventricle sphericity (end diastole) (%)		62.7 ± 1.4		
Ventricle sphericity (end systole) (%)		59.8 ± 2.0		
Myocardial volume (µl)		22.8 ± 6.2		
Ejection fraction (%)		46.8 ± 16.3		
Oxygen consumption rate (ml min^−1^ kg^−1^)				29.5 ± 5.9

#### Echocardiography for axolotl heart modeling and morphometric and functional analysis

Three axolotls (Group A), BM = 17.2 ± 6.3 g, TL = 13.0 ± 1.6 cm, were used for 4D modeling and morphometric analyses of the axolotl heart. Prior to imaging, the animals were anesthetized by immersion in an induction bath of 6 mg l^−1^ propofol (2,6-diisopropylphenol) solution for 30 min and then maintained on 3 mg l^−1^ propofol for the rest of the experiment. The entire cardiac region was imaged with a series of 0.25 mm spaced transversal B-mode ultrasound acquisitions with 250 frames (35–45 frames s^−1^), spanning ~ 3 cardiac cycles pr. acquisition. Reconstructions were made in Amira (version 6.5, FEI SAS, USA), as previously described^20^.

Another four axolotls (Group B), BM = 35.1 ± 4.0 g, TL = 18.4 ± 0.3 cm, were used to measure functional parameters of the heart: Heart rate (*HR*), stroke volume (*SV*), cardiac output (*CO*) and ejection fraction (*EF*). Prior to ultrasound imaging animals were anesthetized by immersion in 200 mg l^−1^ benzocaine (ethyl 4-aminobenzoate) for 30 min. Heart rate was measured in pulsed wave Doppler mode at the outflow tract. Combined myocardium and blood volumes of the heart ventricle at end diastole and end systole were acquired as described above by sweeping the ultrasound transducer across the entire cardiac region to obtain a 4D dataset of the beating heart consisting of at least three cardiac cycles pr. slice. However, as volume estimation does not require the same small step size as modeling, only 9–11 sagittal slices spaced by 1 mm (slice thickness, z) were acquired at this instance. End diastolic and end systolic ventricle volume (myocardium and blood combined) was calculated by tracing the outer surface of the epicardium in these two cardiac phases for each acquired slice and using the formula:

1 $$V\left( {myocardium + blood} \right)_{cardiac\,phase} = z \times \mathop \sum \limits_{i = 1}^{n} A_{i}$$

where *A*_*i*_ is the area of the ventricle cross-section at the *i*th slice and *n* is the total number of slices. Stroke volume was calculated by:

2 $$SV = V\left( {myocardium + blood} \right)_{end\,diastole} - V\left( {myocardium + blood} \right)_{end\,systole}$$

and *CO* was calculated by:

3 $$CO = \frac{SV \times HR}{{BM}}$$

To measure *EF* in the trabeculated heart of the axolotl with no well-defined inner border of the cardiac wall, myocardial volume (*V(myocardium)*) was measured using quantitative histology as described below, and *EF* was calculated using the formula:

4 $$EF = \frac{SV}{{V\left( {myocardium + blood} \right)_{end\,diastole} - V\left( {myocardium} \right)}}$$

#### Ultrasonographic evaluation of flow separation in the axolotl heart

**Figure 1:**
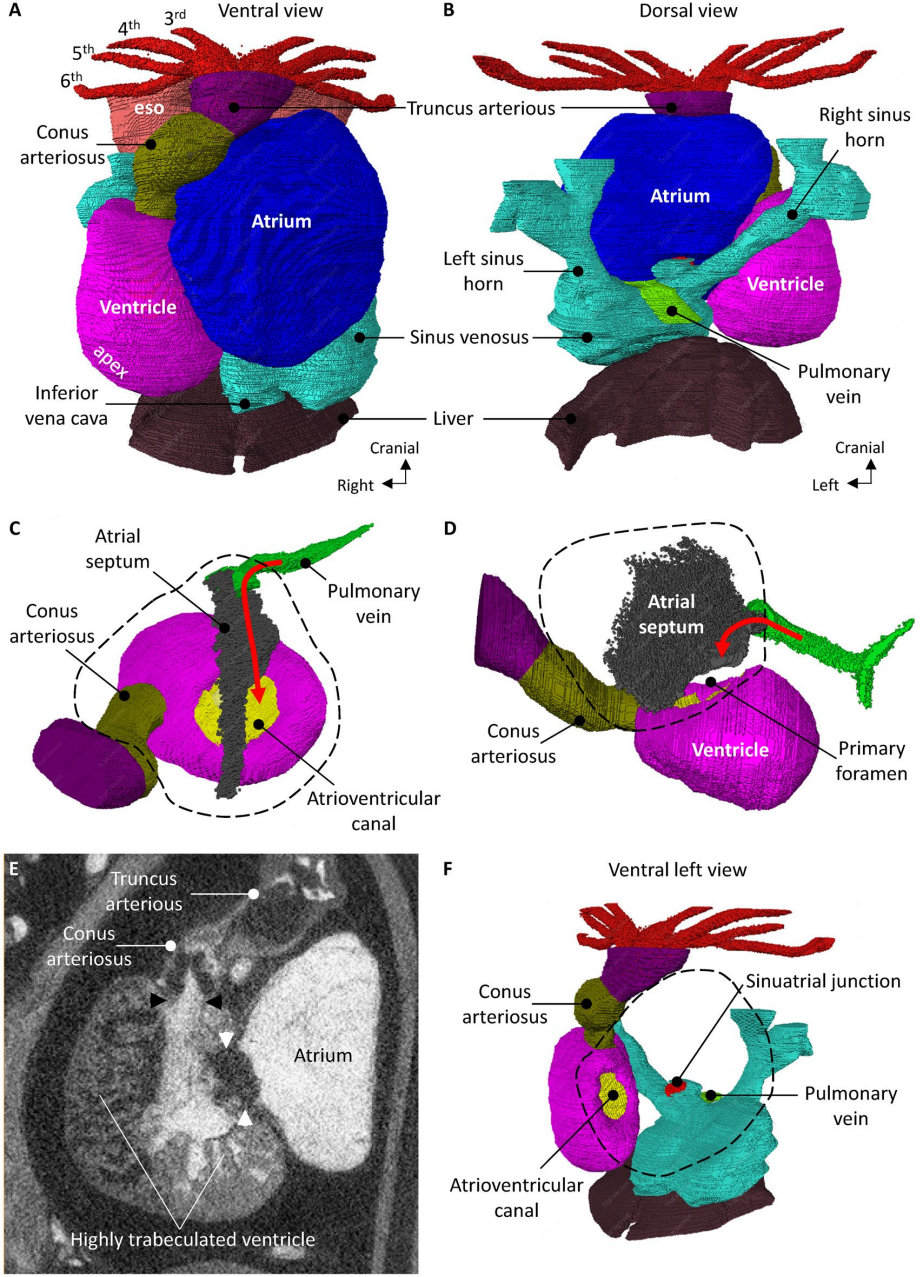
Anatomy of the axolotl heart. (**A**) Ventral view of the in situ axolotl heart showing the principle chambers, namely sinus venosus, atrium (internally divided by an almost complete atrial septum), ventricle and conus arteriosus. The ventricle is positioned on the right and the blunt apex is positioned caudally. The arterial outflow tract is a solitary trunk (truncus arteriosus) with left–right paired channels, giving rise to the third (3rd), fourth (4th), fifth (5th) and sixth (6th) gill arch arteries. There is no diaphragm between the heart and liver. (**B**) Dorsal view, showing the presence of a left sinus horn. Notice that a solitary pulmonary vein connects to the atrium in a position that equals the human embryonic position, i.e. immediately cranial to the sinus venosus and to the left of the sinuatrial junction. (**C**) View of the ventricular base and the atrial septum. The pulmonary vein connects to the left atrium in a primitive position, i.e. close to the ventricular base and next to the atrial septum. (**D**) Left-sided view of the atrial septum, showing a small gap between its leading edge and the atrioventricular canal, i.e. the primary foramen of the atrial septum. (**E**) Image from microCT showing the highly trabeculated state of the ventricle. Notice there is no ventricular septum, there is a solitary atrioventricular canal (white arrowheads) and a common orifice to the outflow tract (black arrowheads). (**F**) Ventral-left view of the heart without the atrium (its outline is indicated by the dashed line). Notice the central position of the solitary atrioventricular canal and the cranial-right position of the outflow tract (conus arteriosus). Such topology resembles a double-inlet left ventricle with double-outlet right ventricle. eso, esophagus.

The same three animals used for 4D modeling (Group A) were used for ultrasonographic evaluation of flow separation in the axolotl heart. Due to the high echogenicity of amphibian blood containing nucleated erythrocytes, isosmotic amphibian Ringer’s solution (112.94 mM NaCl, 2.01 mM KCl, 1.35 mM CaCl_2_, 2.38 mM NaHCO_3_, total osmolarity = 238.71 mOsmol l^−1^) was used as a negative contrast agent appearing dark compared to blood. Following the initial 4D scan, the inflow of the heart was surgically exposed with a small incision and by lifting a portion of the pectoral girdle out of the way. The sinus venosus was non-occlusively cannulated with a PE20 catheter. The ultrasound transducer was placed over the sinus venosus and correct placement of catheter was verified by injecting a small portion of Ringer’s solution resulting in a dark trace within the sinus venosus. The ultrasound transducer was then positioned over the outflow tract with a short axis orientation to transversally image the origin of the eight gill arches (3R/L–6R/L, see Fig. 1). In a long recording of 6800 frames (25 f s^−1^) starting with five cardiac cycles with no infusion for baseline, Ringer’s solution was infused at an increasing rate of 0.01, 0.1, 0.5 and 5 ml min^−1^ for 30 cardiac cycles (only three cardiac cycles for 5 ml min^−1^ infusion) separated each with 30 cardiac cycles pause with no infusion to allow for a complete washout and mixing of previously infused Ringer’s solution. Blood signal change in each gill arch was evaluated by measuring average signal intensity at end diastole in all cardiac cycles in regions of interest (area = 0.063 mm^2^) placed within each gill arch.

#### Visual evaluation of flow separation in the axolotl heart by dye injection

Two axolotls (Group C), BM = 6.6 ± 0.3 g, TL = 9.8 ± 0.4 cm, were used for visual evaluation of flow separation in the heart using Evans Blue injection. Animals were anesthetized by immersion in an induction bath of 6 mg l^−1^ propofol solution for 30 min and then maintained on 3 mg l^−1^ propofol for the rest of the experiment. The entire heart was surgically exposed and the sinus venosus was non-occlusively cannulated with a PE20 catheter. A video (1280 × 720 px^2^, 119.34 frames s^−1^) of the heart region was recorded with a smartphone camera (Samsung Galaxy S6) while a 10.4 mM solution of Evans Blue was infused at 5 µl min^−1^ for 1.68 min reaching a total amount of injected dye of 8.4 µl.

#### Quantitative histology

Immediately following cardiac functional evaluation with echocardiography the hearts of the four axolotls (Group B) were excised, flushed with heparinized (100 units ml^−1^) amphibian Ringer’s solution, blotted on tissue paper, immersed in Tissue Tek optimal cutting temperature medium in a cryomold and snap frozen on a plastic cradle floating on liquid nitrogen. Subsequently, samples were cryosectioned at a 10 µm slice thickness and a 100 or 200 µm spacing between slices to obtain 24–48 equally spaced slices spanning the entire heart. Tissue slices were digitized with a slide scanner and ventricular tissue volume (myocardium + valves) was measured using stereology (point counting) as described by Mühlfeld et al.^21^.

#### Respirometry

Six axolotls (Group D), BM = 63.7 ± 17.3 g, TL = 21.0 ± 2.3 cm, were used to measure oxygen consumption rate (MO_2_) using closed respirometry. Before respirometry, resting conscious heart rate was measured using Eulerian video magnification as described by Lauridsen et al.^22^. A Unisense Clark type oxygen sensor (tip size 25 µm) was calibrated using tempered water bubbled with atmospheric air (20.95% O_2_) or a 100% oxygen gas respectively. Daily barometric pressure and relative humidity was used to calibrate for vapor pressure. Axolotls were fasted for two days prior to respirometry. Closed respirometry was performed at normal housing temperature of 20.6 ˚C in 1.1 l oxygen impenetrable glass containers for two hours with water samples being withdrawn to measure oxygen content just after respirometer closure and again at the end of the experiment.

### Human

#### Double inlet left ventricles

Unoperated patients diagnosed with DILV followed at the Center for Congenital Heart Disease, University Medical Center Groningen, The Netherlands, were identified and studied. ‘Unoperated’ was defined as no prior Fontan procedure (including uni- and bidirectional partial cavopulmonary connection).

The investigation conforms to the principles outlined in the Declaration of Helsinki. The institutional ethics committee approved the conduct of this investigation, and patient informed consent was waived. Patient characteristics were collected retrospectively from medical records and included age, sex, cardiac anatomy and medical history.

#### Data collection

NYHA class, oxygen saturation measured by pulse oximetry, cardio-pulmonary exercise testing (CPET), echocardiography and cardiac magnetic resonance imaging (CMR) data were collected from the most recent clinic visit.

#### Cardio-pulmonary exercise testing

CPET was performed on an upright cycle ergometer. Peak VO_2_ (pVO_2_) was calculated as the mean of the last 30 s during exercise and was indexed for body weight (pVO_2_ indexed). The pVO_2_ as percentage of predicted was calculated using reference values^23, 24^. Adequate performance of CPET was defined as RER > 1.0 in agreement with guidelines for both the pediatric population as well as for adults with heart failure^23, 25^.

#### Cardiac magnetic resonance imaging

CMR of patient 2 was performed on a 1.5 Tesla system (Siemens, Magnetom Avanto, Erlangen, Germany). The CMR protocol included a stack of short-axis slices from the base to the apex of the heart using cine-steady-state free precession with end-expiratory breath holding. Imaging analysis was performed using Qmass (v 7.6.14.0 Medis Medical Imaging Leiden, the Netherlands). The end-systolic and end-diastolic blood volumes were calculated from the endocardial contours; both the volumes of the dominant and hypoplastic ventricle were included for the calculation of ejection fraction. For ease of comparison, mass-specific cardiac output (ml/min/kg) was calculated. For 3D reconstruction Mimics Medical (v 23.0, Materialise, Leuven, Belgium) was used. To assess blood flow magnitude and direction in patient 3, 4D flow MRI on a 3 Tesla system (Philips healthcare, Best, The Netherlands) was performed according to the consensus statement^26^ in a sagittal volume over the heart and aorta (field of view: 350 mm × 350 mm × 117,5 mm), in free breathing with a lung-liver respiratory navigator and with prospective ECG gating. The spatial resolution was 2.5 mm × 2.5 mm × 2.5 mm reconstructed into a voxel size of 1.22 mm × 1.22 mm and slice thickness of 0.89 mm. The temporal resolution was 46 ms (19 timeframes) and a velocity sensitivity (VENC) of 90 cm/s was used. Echo and repetition time were 2.9 and 4.6, respectively, and a flip angle of 10° was used. The raw 4D flow DICOM files were processed in GTFlow (Gyrotools, Zurich, Switzerland) including background phase correction, velocity unwrapping and creation of streamline and pathline images.

#### Echocardiography

Transthoracic echocardiography was performed using a commercially available General Electric ultrasound machine with a 3.5 MHz probe. A standardized protocol was used, which included parasternal, apical, subcostal and suprasternal views.

#### Left ventricles with excessive trabeculation

To compare the ventricular wall anatomy of human and axolotl, we re-examined previously published histological sections of normal and excessively trabeculated human ventricular walls of fetuses^27^ and adults^28^.

### Statistics

All continuous variables are presented as mean (± standard deviation, SD) if parametric or as median (interquartile range) if nonparametric. Categorical data are presented as frequency (percentage of total). For all tests, we used SPSS (version 25, IBM, USA) and we considered P-values less than 0.05 to signify statistical significance.

## Results

### Axolotl and DILV cardiac anatomy

Topographically, the axolotl heart is located behind the pectoral girdle within a pericardial cavity. The lungs are mostly caudal to the heart and there is no diaphragm. In humans, the heart is located in a pericardial cavity behind the sternum, between the lungs, and a diaphragm is present. The axolotl heart comprises the following chambers: the sinus venosus, the left and right atrium, the unseptated ventricle and the myocardial outflow tract called conus arteriosus (Fig. 1A). In embryonic development, the mammal heart also has a sinus venosus^29^ and a conus arteriosus^30^ and electrical activation of which is distinct from that of the atria and ventricles. The human DILV heart comprises the following parts: the left and right atrium, the ventricles (one large, dominant, the other one hypoplastic, connected through a ventricular septal defect) and two arterial outflow tracts. Within the pericardial cavity, in the axolotl the atria are located on the cranial left, whereas the ventricle is on the caudal right. In DILV, the chamber topology is essentially normal with the atria located on the cranial right, and the ventricles on the caudal left. In the axolotl the ventricular apex is very rounded and points caudal-right rather than caudal-left as in human (Fig. 1A).

In the axolotl, the systemic venous blood drains to the right atrium via the caval veins. The left caval vein resembles the human coronary sinus with a persistent left sinus horn (Fig. 1B)^29^. In DILV, the systemic venous blood drains to the right atrium via the caval veins and coronary sinus. In the axolotl, pulmonary venous blood drains to the heart by a solitary pulmonary vein (Fig. 1B). In DILV, the pulmonary veins may be normal and four of them drain into the left atrium. In axolotls there is a well-developed primary atrial septum only, there is no second septum and oval fossa, and the primary atrial septum is not fully merged with the atrioventricular valve (Fig. 1C,D)^31^. In DILV, the atrial septum completely merges with the atrioventricular valves. In axolotls, there is a common atrioventricular orifice with a single valve that consists of four cusps. In DILV, there are two separate atrioventricular orifices that both connect to the dominant left ventricle^32^.

The axolotl ventricle is a highly trabeculated chamber with a sponge-like appearance (Fig. 1E). In its center, the sponge is aggregated into a few parallel sheets but there is no obvious ventricular septum (Fig. 1E)^33^. The atrioventricular orifice is located approximately in the center of the ventricular base, or slightly to the left. In DILV, the dominant, large ventricle is morphologically left, with fine criss-crossing trabeculations^34^. Almost always a small, hypoplastic ventricle of right morphology with coarse trabeculations is present and it is connected to the left ventricle by means of a ventricular septal defect (VSD). Together the two ventricles function as a single ventricular unit^10^. There is variability in the exact location of the hypoplastic ventricle. It is always located on the anterior side of the left ventricle, but can be either left, right, or medial^35^.

In the axolotl, the solitary outlet, conus arteriosus, is located on the right in the ventricular mass (Fig. 1F). The conus wall comprises myocardium and its contraction is delayed compared to that of the ventricle^11, 12^. A valve guards the conoventricular orifice. In the axolotl, the conus lumen comprises a systemic and a pulmonary channel which are separated by the so-called spiral valve. From the conus emerges the arterial truncus arteriosus which has eight channels, one for each of the left–right paired gill arches (3rd, 4th, 5th, and 6th) with arches 3 to 5 supplying the three paired gills in the axolotl (Fig. 1A,B). Similarly, the 6th gill arch, together with the efferent gill artery of the third and most caudal gill (supplied by the 5th gill arch), supply the axolotl lung (Supplementary material S1). There is a cono-truncal valve. The numerous differences between the adult hearts of axolotl and human aside, the axolotl heart has a much greater likeness to the human embryonic heart (Supplementary material S2).

In DILV, usually there are two distinct outlets, the pulmonary artery and the aorta. There is variability with regards to the ventriculoarterial connection. It can be either concordant, meaning the aorta arises from the left ventricle and the pulmonary artery from the right ventricle, or discordant, with the aorta arising from the right ventricle and the pulmonary artery from the left ventricle, or, in rare cases, both the aorta and the pulmonary artery can arise from the left or right ventricle, or a single outlet of the heart, either with a common arterial trunk, or with either an atretic aortic or pulmonary trunk^35^. A discordant ventriculoarterial connection is most commonly encountered^1, 35^. Pulmonary stenosis can be present on the valvar, subvalvar or pulmonary artery level. Generally speaking, DILV hearts are a very heterogenous group with variable anatomical characteristics.

### Examples of DILV heart anatomy

Three DILV patients that had never undergone the Fontan procedures were identified. For a complete overview of anatomical and functional characteristics see Table 2.

**Table 2:** Overview of the DILV patients.

	Patient 1	Patient 2	Patient 3
Age (years)	52	16	56
Height (cm)	150	175	163
Weight (kg)	80	67	68
Sex	Female	Female	Female
**Anatomic characteristics**			
Pulmonary vascular protection	Pulmonary stenosis	Restrictive VSD	No (Eisenmenger Syndrome)
Location hypoplastic ventricle	Right	Right	Left
Ventriculoarterial connection	Concordant	Concordant	Discordant
VSD restrictive	No	Yes	No
**Functional characteristics**			
NYHA class	II	I	II
Heart rate at rest (beats min^−1^)	90	63	62
End-diastolic volume (ml; ml m^−2^)	204 (104)	236 (127)	261 (150)
End-systolic volume (ml; ml m^−2^)	92 (47)	99 (53)	103 (60)
Ejection fraction (%)	55	58	60
Stroke volume (ml)	112	137	158
Cardiac output (l min^−1^)	10.1	8.6	9.8
Mass specific cardiac output (ml min^−1^ kg^−1^)	126.3	128.4	144.1
Cardiac index (l min^−1^ m^−2^)	5.1	4.6	5.7
Systemic oxygen saturation at rest %	91	100	87
Peak oxygen consumption (ml O_2_ min^−1^ kg^−1^; % predicted)	6.3 (25)	31 (67)	13 (57)
RER	1.01	1.10	1.03
**Laboratory measurements**			
Hemoglobin (mmol l^−1^)	13.9	8.7	12.9
Hematocrit (l l^−1^)	0.68	0.39	0.65

#### Patient 1

There was no atrial septal defect, both atrioventricular valves were connected to the dominant left ventricle, the right-sided atrioventricular valve was straddling. The hypoplastic ventricle was located on the right side of the dominant ventricle. The VSD was large and non-restrictive. The ventriculoarterial connection was concordant. There was presence of congenital subvalvar and valvar pulmonary stenosis with a gradient of 75 mmHg on echocardiography.

#### Patient 2

**Figure 2:**
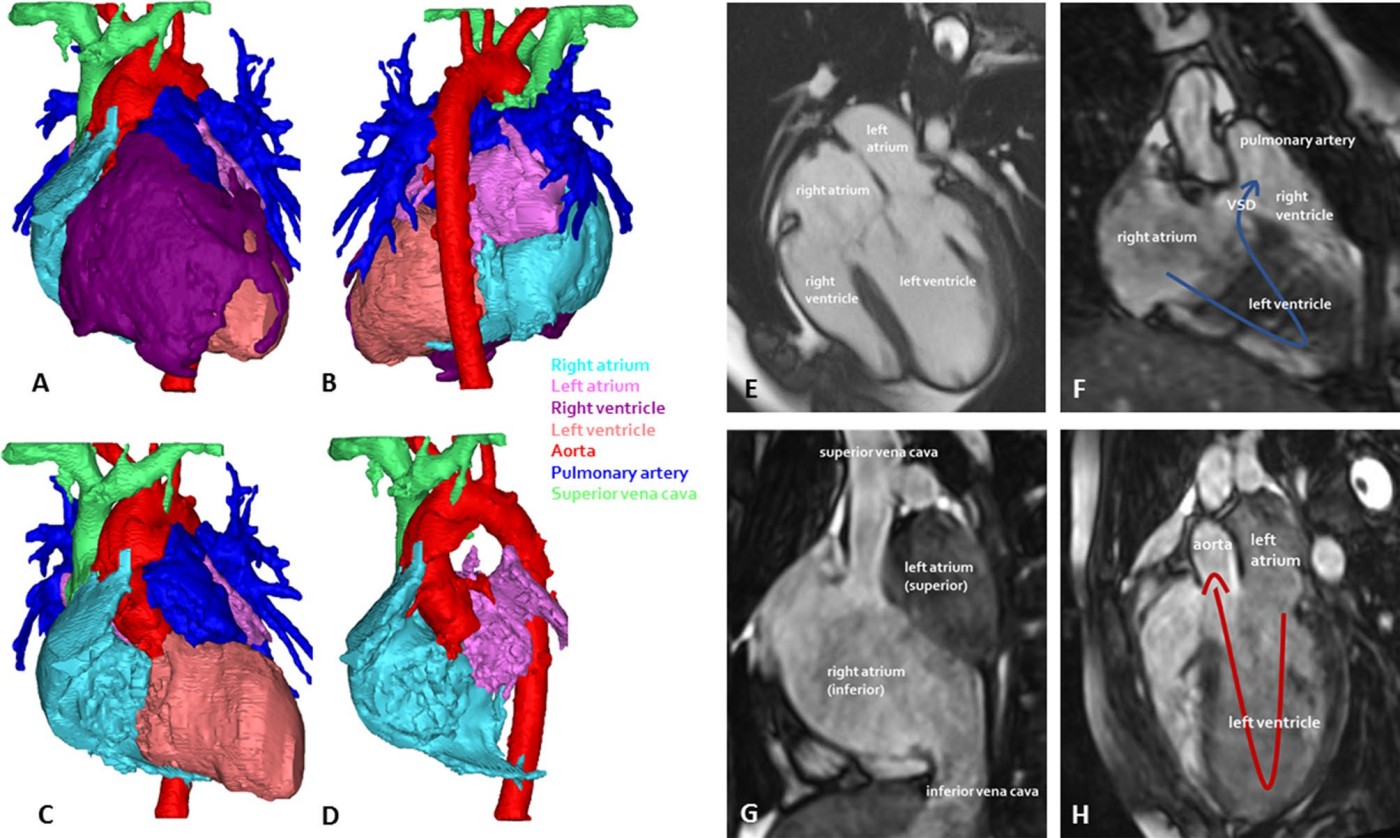
Anatomy of double inlet left ventricle patient 2. (**A**) Anterior view, complete. (**B**) Posterior view, complete. (**C**) Anterior view, right ventricle removed. (**D**) Both ventricles removed, appreciate anterior/posterior relationship of atria. (**E**) 4-chamber view. (**F**) Pathway of oxygen-poor blood stream. (**G**) Superior-inferior relationship of the atria. (**H**) Pathway of oxygen-rich blood stream. VSD, ventricular septal defect.

There was no atrial septal defect, the left atrium was located superior to the right atrium (Fig. 2). Both atrioventricular valves were connected to the dominant left ventricle. The right-sided atrioventricular valve was straddling and overriding (30% right, 70% left). The hypoplastic ventricle was located on the right side of the dominant ventricle. The VSD was small and restrictive with a peak velocity of 4.7 m/s. The ventriculoarterial connection was concordant. There was no pulmonary stenosis, however, the pulmonary vascular bed was protected from volume overload due to the restrictive nature of the VSD.

#### Patient 3

**Figure 3:**
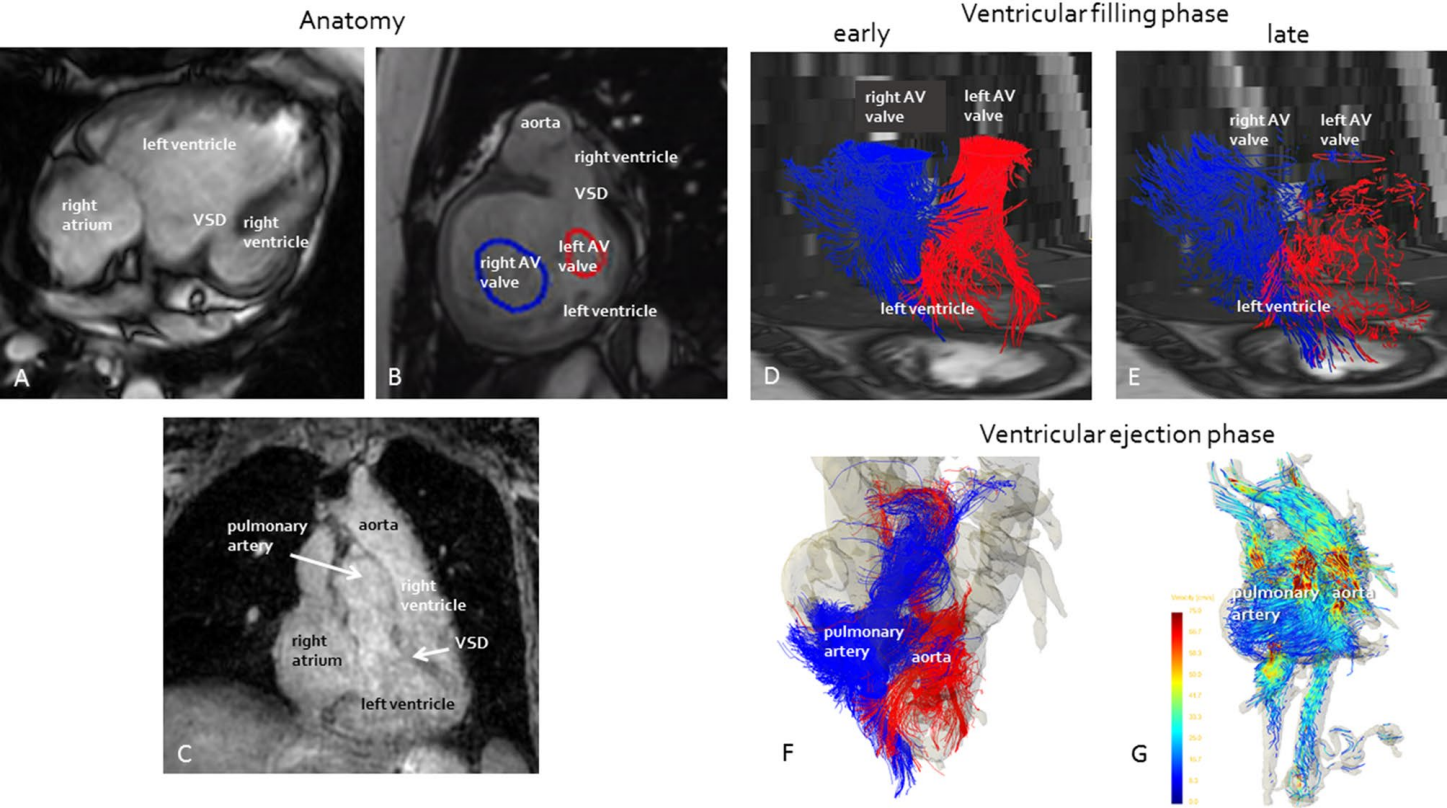
Anatomy and 4D streaming analysis of double inlet left ventricle patient 3. (**A**) 3-chamber view. (**B**) Sagittal view. (**C**) Coronal view. (**D**) Early ventricular filling phase depicting oxygen-poor (blue) blood and oxygen-rich (red) blood streams entering the left ventricle through the right and left atrioventricular valves respectively. (**E**) Late ventricular filling phase depicting oxygen-poor (blue) blood and oxygen-rich (red) blood streams entering the left ventricle through the right and left atrioventricular valves respectively. (**F**) Ventricular ejection phase depicting oxygen-poor (blue) blood and oxygen-rich (red) blood streams entering the pulmonary artery and aorta respectively. Oxygen-poor blood is not ejected in the aorta, blood ejected into the pulmonary artery seems partially oxygen-rich and oxygen-poor. (**G**) Ejection of blood in aorta (left) and pulmonary artery (right).

There was no atrial septal defect. Both atrioventricular valves were connected to the dominant left ventricle. The hypoplastic right ventricle was located on the left side of the dominant ventricle. The VSD was large and non-restrictive. The ventriculoarterial connection was discordant (Fig. 3). There was no pulmonary stenosis and the patient had developed Eisenmenger syndrome as a result.

### Cardiac function and oxygen consumption in the axolotl and DILV

Functional parameters of the axolotl are reported in full in Table 1. Heart rate in resting axolotl was 21.7 ± 4.1 beats min^−1^ whereas under anesthesia it was 27.3 ± 6.4 beats min^−1^ using propofol and 43.3 ± 0.5 beats min^−1^ using benzocaine. Stroke volume under propofol and benzocaine anesthesia was 33.1 ± 13.1 µl and 41.3 ± 8.5 µl, respectively, and CO under propofol and benzocaine anesthesia was 52.3 ± 13.7 ml min^−1^ kg^−1^ and 51.6 ± 14.3 ml min^−1^ kg^−1^, respectively. Ejection fraction under benzocaine anesthesia was 46.8 ± 16.3%. Oxygen consumption rate of unanesthetized animals was 29.5 ± 5.9 ml O_2_ min^−1^ kg^−1^.

Functional parameters of the DILV patients are reported in Table 2. Ejection fraction was greater than 50% in all three patients. Systemic oxygen saturation at rest varied between patients, with the lowest being 87% in patient 3 with Eisenmenger syndrome and the highest being 100% in patient 2. Peak oxygen consumption during cardiopulmonary exercise testing was severely limited in patient 1 and 3, and mildly limited in patient 2. NYHA functional class was I or II in all three patients.

### Flow separation in the axolotl and DILV

**Figure 4:**
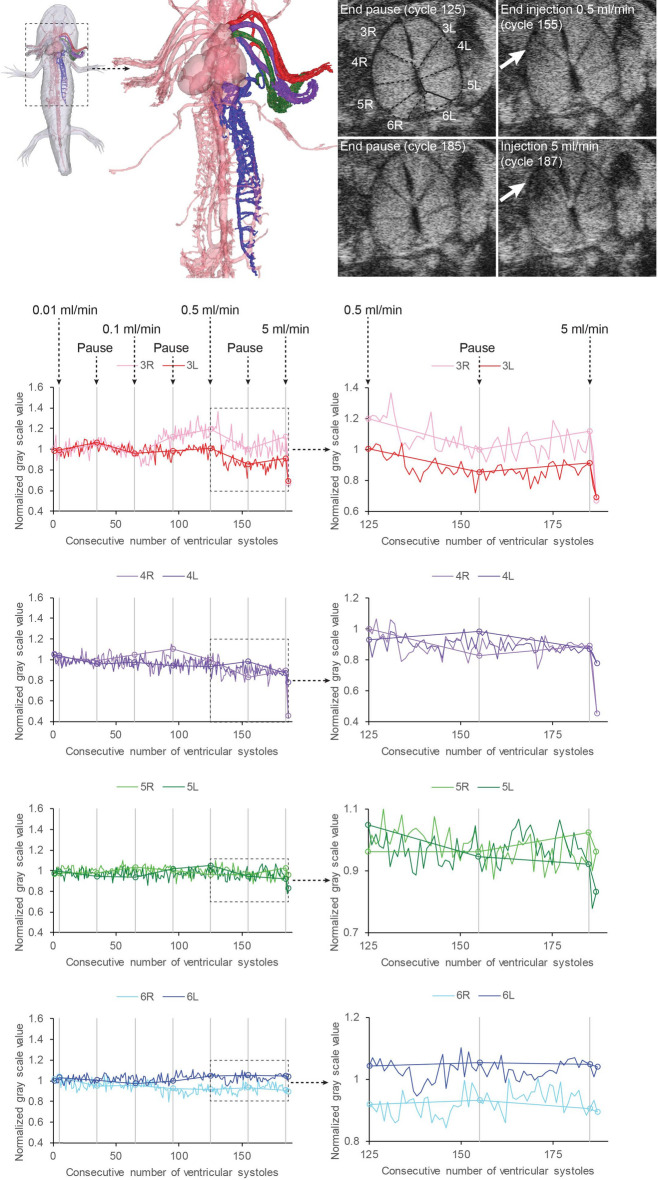
Cardiac flow separation in the axolotl demonstrated with ultrasonography. Top left: Anatomical model of the gill arches in the axolotl (3rd arch red, 4th arch purple, 5th arch green, 6th arch blue). Top right: Ultrasonographic transversal cross sections in the outflow tract at four time points in the injection series of negative contrast agent (amphibian Ringer’s solution). Notice the signal decrease (arrows) as diluted blood perfuse the three ventral gill arches after 30 and 3 cardiac cycles of contrast agent infusion at 0.5 and 5 ml min^−1^ respectively. Main part: Signal traces in right and left arches over time. Traces on the right are magnifications of the last 63 cardiac cycles with a high rate of infusion of contrast agent. Notice the final signal decrease in the 3rd, 4th, and 5th gill arches supplying the gills but not in the 6th gill arch supplying the lungs indicating a lack of complete mixing of blood in the heart as hypointense contrast agent is injected into the sinus venosus at a high flow rate (5ml min^-1^).

Visual inspection on the exposed heart region in axolotls of group C during venous injection of Evans Blue indicated that incoming dye from the sinus venosus and right atrium did not mix completely in the ventricle (see video in Supplementary material S3). This observation was confirmed in the ultrasonographic evaluation (Fig. 4). Infusion of amphibian Ringer’s solution at low flow rates did not produce detectable contrast differences at the level of the outflow tract, but injection at a flow rate of 0.5 ml min^−1^ and in particular at 5 ml min^−1^ produced a short decrease in signal from the blood in the three arches supplying the gills (arch 3–5). The 6th arch supplying the lungs, however, did not receive diluted blood showing some capacity for flow separation in the unseptated ventricle (compare the signal in gill arches 3, 4 and 5 with the signal in gill arch 6 for the last cardiac cycles in the recording in Fig. 4). 4D flow MRI of patient 3 showed that the two disparate blood stream entering and exiting the ventricles remain partially separated (Fig. 3, and videos in Supplementary material S4). The 3D MRI of patient 2 showed that there is an inferior/superior relationship of the two atria, whereby oxygen-poor blood from the right atrium may enter the ventricle on the right side and take the outer bend through the VSD into the hypoplastic right ventricle and pulmonary artery, whereas oxygen-rich blood from the left atrium may enter the ventricle on the left side and take the inner bend straight to the aorta (Fig.2).

The 3D MRI of patient 2 showed that there is an inferior/superior relationship of the two atria, whereby oxygen-poor blood from the right atrium may enter the ventricle on the right side and take the outer bend through the VSD into the hypoplastic right ventricle and pulmonary artery, whereas oxygen-rich blood from the left atrium may enter the ventricle on the left side and take the inner bend straight to the aorta (Fig. 2).

### Left ventricles with excessive trabeculation compared to the axolotl ventricle

**Figure 5:**
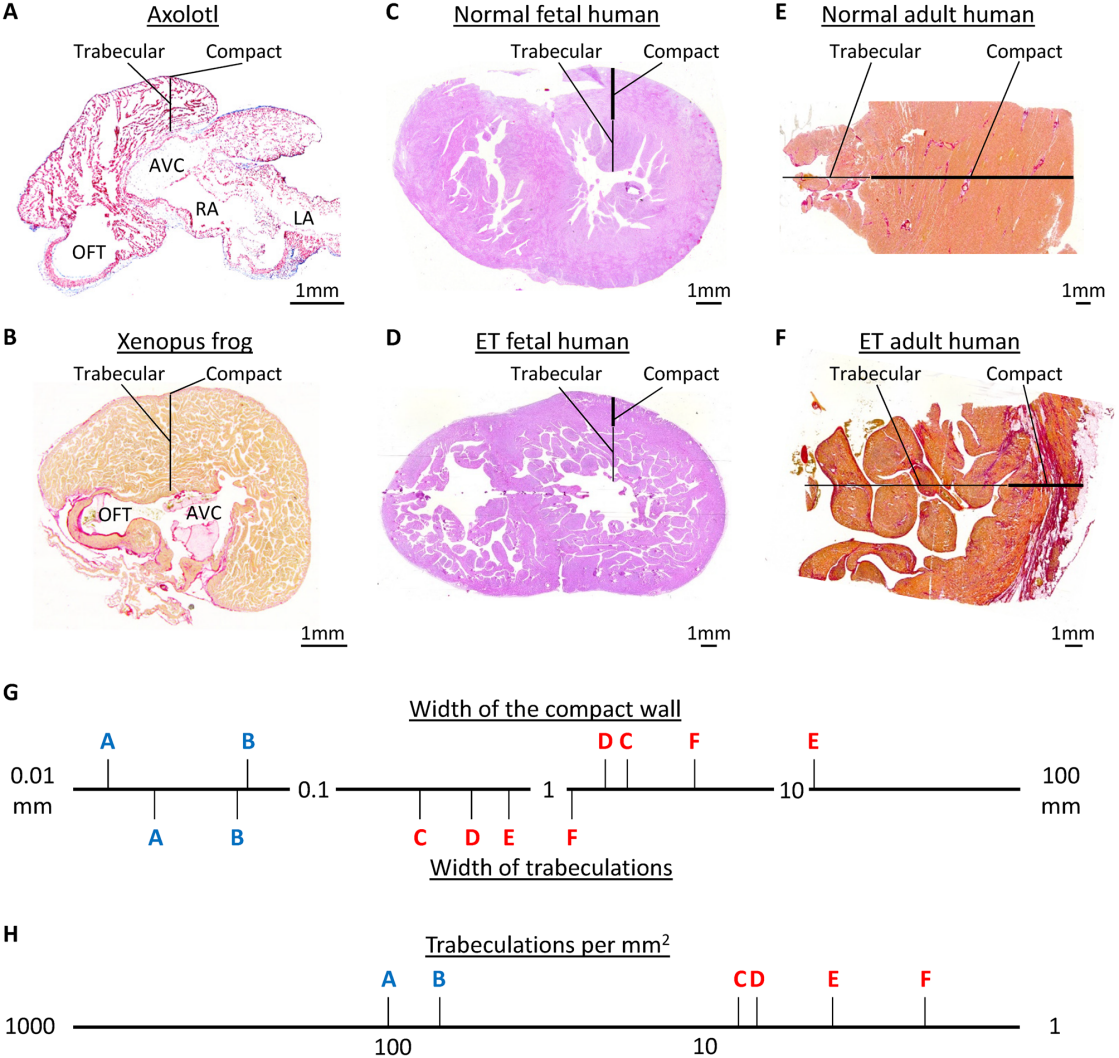
Ventricular wall composition in amphibians and human with normal and excessively trabeculated left ventricle. (**A**,**B**) Transverse section of the ventricular base of an axolotl (**A**) and a Xenopus frog (**B**), showing that the ventricle is highly trabecular and the compact wall is extremely thin. It can also be seen that the solitary atrioventricular canal (AVC) is left-sided and the solitary outflow tract (OFT) is right-sided. (**C**) Transverse section of the human ventricles at gestational week 20. This heart is not much bigger than the Xenopus heart (**B**), but the compact wall is much thicker and the trabeculations are much fewer and greater in size. (**D**) Transverse section of a case of fetal human excessive trabeculation at gestational week 20. Compared to the heart shown in (**C**), the compact wall is thinner and the trabeculations are more numerous, but this heart is still much different from the amphibian hearts (**A**–**B**). (**E**) Transmural histology of a normal left ventricle of an adult human, showing a thick compact wall and a few trabeculations. (**F**) Transmural histology of a case of left ventricular excessive trabeculation of an adult human. Compared to the heart shown in (**E**), the compact wall is thinner and the trabeculations are more numerous, but this wall composition is still much different from that of the amphibian hearts (**A**–**B**). (**G**) Measurements of the width of the compact wall (above the horizontal axis) and trabeculations (below the horizontal axis), shown on a logarithmic scale. Letters refer to the images (**A**–**F)** on which the measurements were done. (**H**) Counting of trabeculations per mm^2^ on images (**A**–**F**) showing that the amphibians have a 1–2 orders greater number of trabeculations per area. Staining is Masson’s trichrome in (**A**), picro-sirius red in (**B**, **E** and **F**), and hematoxylin–eosin in (**C** and **D**). The sections shown in (**C**,**D)** were part of the data published in^27^.

We measured the thickness of the compact layer and trabeculations on seven histological sections from four axolotls (Group B) (Fig. 5A). The compact layer was on average 15.7 ± 2.9 µm thick which was significantly thinner than the average trabecular width of 19.7 ± 3.1 µm (paired t-test = 0.004, n = 7). In the *Xenopus* frog, another common aquatic amphibian experimental organism, the wall morphology was similar (Fig. 5B). Fetal human hearts of around 20 weeks of gestation are not much bigger than those of the amphibians, yet the compact wall is much thicker and the trabeculations are much fewer and thicker (Fig. 5C). Even if the ventricles are excessively trabeculated, the compact wall is much thicker than in the amphibians. Also, although the trabeculations are numerous, they are still much bigger and far fewer than in the amphibians (Fig. 5D). In the adult heart, irrespective of whether the left ventricle has a normal wall or an excessively trabeculated wall, the wall architecture is much less trabeculated than that of amphibians (Fig. 5E,F). Measurements of widths of the compact wall and the trabeculations reveal more than an order of magnitude greater widths in human (Fig. 5G). Accordingly, the number of trabeculations per area can be a couple of orders of magnitude larger in amphibians (Fig. 5H).

## Discussion

The axolotl has attributes that could make it an interesting model for human DILV and ET. Both in the axolotl as well as in DILV, disparate blood flows can stay separated in a single ventricle through streaming. This may be possible through an interplay of various anatomical characteristics that lead to a separation with flows staying separated on either side and being directed along the inner and outer bend of the single ventricle. For the axolotl, this is the natural situation, whereas for humans, albeit not ideal, it can aid in facilitating survival for those born with functionally univentricular hearts, and, in the most favorable cases, may allow for a higher life expectancy and better quality of life than those observed in Fontan patients. Differences were found with regards to anatomy, most importantly, the axolotl having a common ventricular inlet and outlet, whereas in DILV, there are two separate inlets and outlets. Moreover, it was established that the trabeculations found in ET are closer to the normal human setting than to the axolotl setting. A few case series of DILV survivors are described in the literature so far^7–9^ and cardiac imaging studies of single ventricle amphibians have been conducted^13^, however, this is the first study to directly compare human functionally ventricular hearts to those of the axolotl. Analyses of blood gasses^36–38^, injection of X-ray contrast^39^, and UV-light activated fluorescence^12^ have been used previously to show degrees of streaming in the salamander and anuran amphibian ventricles. Here, we used micro-echocardiography for visualization of streaming in the axolotl single ventricle with Ringer’s solution as a contrast agent which should be innocuous albeit a substantial injected volume was necessary to get good visualizations. Furthermore, this is the first study to use 4D MRI for visualization of streaming in a human DILV without Fontan circulation.

### Cardiac function and oxygen consumption

Cardiac function in the axolotl is known to be affected by the use of anesthetics^22, 40^. The range of values in this study falls well within previously reported heart rate for awake and anesthetized axolotls^22, 40^, generally showing a subtle tachycardia effect of propofol and a more pronounced effect of benzocaine. Resting heart rate in the axolotl was much lower than in DILV (and more general, human hearts). Cardiac output of the axolotl under anesthesia, when corrected for body mass, is similar to that in DILV patients at rest. Because mass-specific CO is dependent on body mass, the values tend to be greater in lighter animals. The fact that the values for CO are comparable in human and axolotl could thus be accounted to a chance effect of the sizes (as well as the endo- vs. ectothermic state) of these two species. The ejection fraction in the axolotl reported here is lower than that in DILV patients, however this may reflect a short and incomplete contraction of the ventricle during benzocaine anesthesia, which can be observed when comparing to echocardiography on awake animals. In amphibians, the highly trabeculated ventricle feed the systemic circulation with blood pressures that are much lower than in human, also DILV, with a mean systemic arterial blood pressure of 20–30 mmHg^41^. Oxygen consumption in the ectothermic axolotl at 20.6 °C is lower than in endothermic healthy human, but comparable to DILV patients.

### Streaming and anatomy

Streamline flows as those entering into the ventricle do not have an intrinsic tendency to mix unless forced by turbulence^42^. This study showed that in the single ventricle of the axolotl and in DILV, favorable streaming, meaning little-to-no mixing of oxygen-rich and oxygen-poor blood, is possible. The possibility of favorable streaming has previously been acknowledged^43–45^. It offers an explanation for why certain DILV patients have high systemic oxygen saturations even though both oxygen-rich and oxygen-poor blood enter the same ventricle. Certain anatomical constitutions in both DILV patients as well as the axolotl may facilitate this favorable streaming. In the axolotl, the atria are located on the left, whereas the ventricle and the conus arteriosus is located on the right. We postulate that the blood from one atrium, due to its location, will stay on one side, whereas blood from the other atrium will stay on the other side, leading to streamlining in the ventricle up to the point where the blood flows enter the outflow tract. In DILV, there is more variance with regards to the spatial relationship of the inflow-and outflow tracts^35^. Ideally, similar to the axolotl, the atria and atrioventricular connection are positioned in a way that blood from one atrium may enter the ventricle and stay on one side, taking the outer bend toward the correct outflow tract, whereas blood from the other atrium may enter the ventricle on the other side and take the inner bend toward the correct outflow tract. This configuration was seen in patient 2, who has favorable streaming with normal systemic oxygen saturation. The presence or absence of pulmonary vasculature protection^7^ can also influence streaming. DILV patients who do not have restriction of pulmonary blood flow inevitably develop Eisenmenger syndrome (patient 3). Although it was demonstrated that blood flows are also partially separated, patient 3 has lower oxygen saturations due to right-left shunting. When the pulmonary vasculature is protected by pulmonary stenosis, as in patient 1 and 2, oxygen saturations are higher.

As opposed to DILV, there is no complete separation on the atrial level in the axolotl, and there is a common atrioventricular valve instead of two separate inlets. Furthermore, the axolotl has a common ventricular outlet through the conus arteriosus, whereas in DILV there are two outlets. Another difference is the trabeculation pattern. We found that the axolotl ventricular trabeculations are much smaller in size and much greater in number than in human, even when compared to ET. The trabeculations in ET resembles the normal human setting in a higher degree than the axolotl setting.

This is a small study that focused on qualitative evaluation and comparison. The results and conclusions are therefore of a somewhat theoretical nature. Especially with regards to possible treatment options and alternatives to the Fontan circulation, larger-scale studies and quantitative analyses are needed in order to find the best, individualized approach for each patient.

This study shows that it is possible for DILV patients to survive and live without Fontan circulation. Although their exercise tolerance was reduced in varying degrees, these patients had a preserved NYHA class and heart function. This can be seen in contrast to patients with a Fontan circulation, where long-term longevity and quality of life is limited due to the un-physiological character of the Fontan circulation^46–49^.

## Conclusion

This is the first study comparing human survivors of DILV and ET to the axolotl heart and circulation. The most important observations are that ET resembles the human setting more than that of the axolotl, and that both in the axolotl as well as in human DILV, disparate blood flows from the two atria can stay separated in a single ventricle through streaming. This study lays the groundwork for future studies focusing on identifying those selected DILV patients that might benefit from non-Fontan palliation, for example by means of a form of pulmonary artery banding.

## Supplementary Information

Supplementary Information 1.

Supplementary Information 2.

Supplementary Video 1.

Supplementary Information 3.

Supplementary Information 4.

## Data Availability

The datasets generated during and/or analysed during the current study are available from the corresponding author on reasonable request.
