# Supplementary figures and images for: Opportunities and short-comings of the axolotl salamander heart as a model system of human single ventricle and excessive trabeculation
Source: Sci Rep. 2022 Nov 28;12:20491. doi: 10.1038/s41598-022-24442-9 (PMC9705478; doi:10.1038/s41598-022-24442-9)

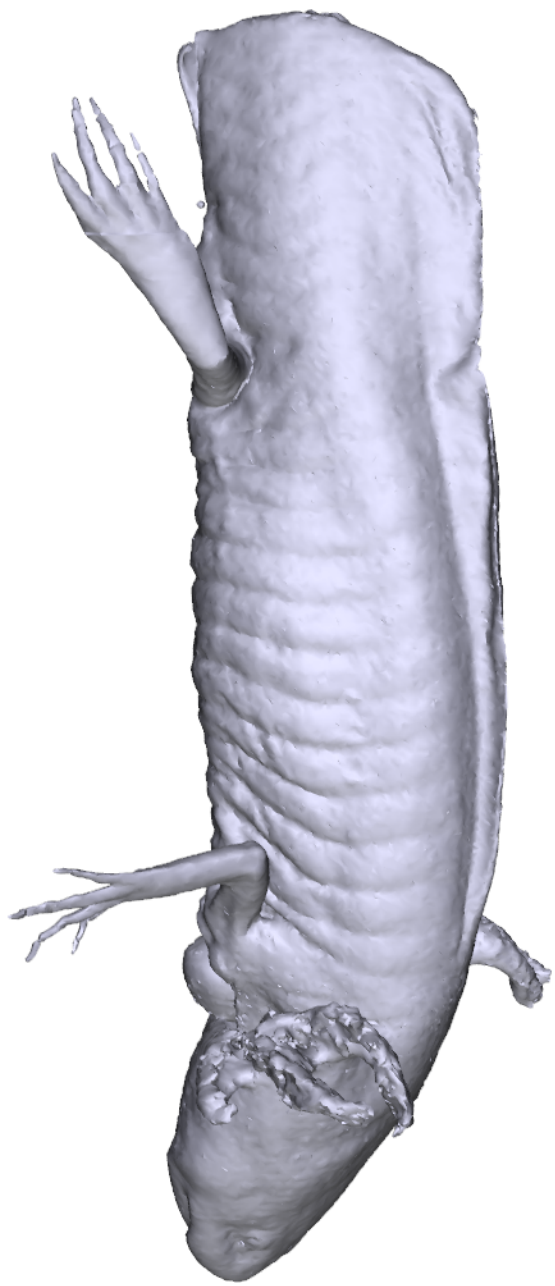

Supplement: Supplementary file 1 — Supplementary Information 1. [file 41598_2022_24442_MOESM1_ESM.pdf]

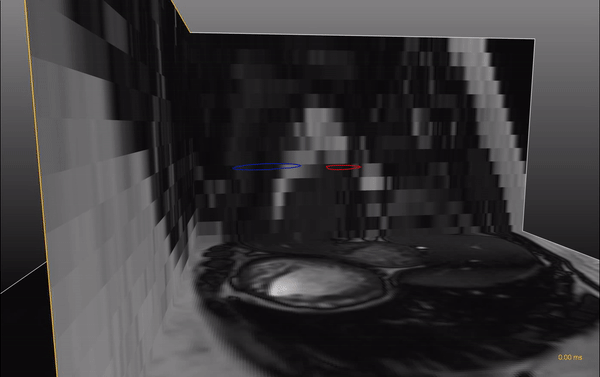

Supplement: Supplementary file 4 — Supplementary Information 3. [file 41598_2022_24442_MOESM4_ESM.gif]

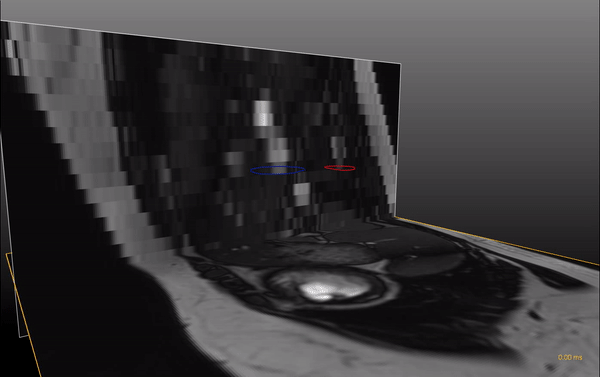

Supplement: Supplementary file 5 — Supplementary Information 4. [file 41598_2022_24442_MOESM5_ESM.gif]
