# Supplementary material for: Opportunities and short-comings of the axolotl salamander heart as a model system of human single ventricle and excessive trabeculation
Source: Sci Rep. 2022 Nov 28;12:20491. doi: 10.1038/s41598-022-24442-9 (PMC9705478; doi:10.1038/s41598-022-24442-9)
Supplement: Supplementary file 2 — Supplementary Information 2. [file 41598_2022_24442_MOESM2_ESM.docx]

**Supplementary material S2 - adult axolotl hearts compared to embryonic human hearts**

Many features of the formed axolotl heart resemble the human embryonic heart. In formed axolotl hearts as in human embryonic hearts, pulmonary venous blood drains to the heart by a solitary pulmonary vein (1,2). In the axolotl, a well-developed primary atrial septum is present, only it is not fully merged with the atrioventricular valve (3). This setting is largely similar to the embryonic human setting just before the closure of the primary atrial foramen (2). In the axolotl there is a common atrioventricular orifice with a single valve that consists of four cusps and this setting is approximately similar to that of embryonic human before the dorsal and ventral atrioventricular cushion have merged (2). The axolotl ventricle is a highly trabeculated chamber with a sponge-like appearance. Such chamber architecture is also found in embryonic human hearts (4). The atrioventricular orifice is located to the left in the ventricular base which resembles the embryonic human setting before the formation of the tricuspid valve (4). In the axolotl, the solitary outlet, conus arteriosus, is located on the right in the ventricular mass. The conus wall comprises myocardium and its contraction is delayed compared to that of the ventricle. A valve guards the conoventricular orifice. A conus-like structure temporarily appears in the human embryo, only it gets internalized in later stages in the right ventricle and contributes to the ventricular septum (5,6). In the axolotl, the conus lumen comprises a systemic and a pulmonary channel which are separated by the so-called spiral valve. From the conus emerges the arterial truncus arteriosus which has eight channels, one for each of the left-right paired gill arches (3^rd^, 4^th^, 5^th^, and 6^th^) with arches 3 to 5 supplying the three paired gills in the axolotl (Fig. 1A-B). These channels are not formed in the human embryo, but the equivalent of the 3^rd^, 4^th^, and 6^th^ gill arches are formed with the 6^th^ gill arches giving rise to the left and right pulmonary trunk (7,8).

References

1. Webb S, Kanani M, Anderson RH, Richardson MK, Brown NA. Development of the human pulmonary vein and its incorporation in the morphologically left atrium. Cardiol Young. 2001 Nov;11(6):632–42.

2. Jensen B, Wang T, Moorman AFM. Evolution and Development of the Atrial Septum. Anat Rec (Hoboken). 2019 Jan;302(1):32–48.

3. Lewis ZR, Hanken J. Convergent evolutionary reduction of atrial septation in lungless salamanders. J Anat. 2017 Jan;230(1):16–29.

4. Faber JW, Hagoort J, Moorman AFM, Christoffels VM, Jensen B. Quantified growth of the human embryonic heart. Biol Open. 2021 Feb;10(2).

5. Kelly RG, Brown NA, Buckingham ME. The arterial pole of the mouse heart forms from Fgf10-expressing cells in pharyngeal mesoderm. Dev Cell. 2001 Sep;1(3):435–40.

6. Sizarov A, Ya J, de Boer BA, Lamers WH, Christoffels VM, Moorman AFM. Formation of the building plan of the human heart: morphogenesis, growth, and differentiation. Circulation. 2011 Mar;123(10):1125–35.

7. Poelmann RE, Gittenberger-de Groot AC. Development and evolution of the metazoan heart. Dev Dyn an Off Publ Am Assoc Anat. 2019 Aug;248(8):634–56.

8. Sizarov A, Lamers WH, Mohun TJ, Brown NA, Anderson RH, Moorman AFM. Three-dimensional and molecular analysis of the arterial pole of the developing human heart. J Anat. 2012 Apr;220(4):336–49.
